# Commencement Exercises of the College of Physicians and Surgeons of Atlanta

**Published:** 1907-06

**Authors:** 


### COMMENCEMENT EXERCISES OF THE COLLEGE OF PHYSICIANS AND SURGEONS OF ATLANTA.

At the annual commencement exercises of the college of physicians and surgeons held April 27th, 37 young doctors received their diplomas.

The address of the evening was delivered by Hon. Robt. C. Allston. Dr. W. S. Elkin, the dean, reported some interesting facts regarding the progress of the college and the splendid new buildings which have been recently acquired and greatly add to the facilities for giving instruction in the various departments. The donation of $10,000 by Andrew Carnegie has been raised to $25,000. The faculty is composed of 41 professors and instructors, some of whom are among the best known physicians in the city. There were 200 students in the medical department during the past session.

The following members of the senior class received their diplomas:

Wilson Pruitt Allen, Georgia; Daniel Clements Alsobrook, Georgia; John Leon Bell, Georgia; Wilmer Cortez Box, Alabama; Stewart Dixon Brown, Georgia; Jesse Edward Coates, Mississippi; Ariel Cook, Jr., Georgia; William Shelley Cook, Georgia; Virgil Cannon Cook, Georgia; William Earnest DeLaPerriere, Georgia; Ramon de Poo Cue, Cuba; John Alexander Dowd, North Carolina; Addie Moran Edwards, North Carolina; Charles Campbell Giddens, Georgia; William Madison Girtman, Georgia; David Franklin Harwell, Georgia; Jake Edwin Haslam, Jr., Georgia; Oliver Burdett Hicks, Louisiana; Gaston Bailey Justice, North Carolina; Chester Overton Middlebrooks, Georgia; Edward Monroe Mc Donald, Georgia; Benjamin Amon McManus, South Carolina; William Daniel Nobles, Florida; Frank Marcellus Nolan, Georgia; Claude Clifford Pearce, Alabama; William Houston Quillian, Georgia; Walter Percy Rhodes, Texas; John Whitehead Seidell, Georgia; Edward Cooper Smith, Georgia; James Parker Stallworth, Alabama; Walter Kenneth Stewart, Georgia; William Aaron Strickland, South Carolina; John Victor Tate, South Carolina; Glenn Lazarus Todd, Mississippi; John Cox Wall, Georgia; Nicholas Aaron Wheeler, Alabama.

The honor men of the class who were presented with certificates of proficiency were: Daniel Clements Alsobrook, Edward C. Smith,

Charles C. Giddings, Walter K. Stewart and John C. Wall, all from Georgia. Those who received honorable mention were. C. C. Pearce, of Alabama; J. E. Coates, of Mississippi, and E. M. McDonald, of Georgia.

Allied with this institution are: The dental department with an enrollment of 691 students, and the pharmaceutical department with 144, making a grand total of 513 students in the three departments.

